# Effects of different vibration frequencies on muscle strength, bone turnover and walking endurance in chronic stroke

**DOI:** 10.1038/s41598-020-80526-4

**Published:** 2021-01-08

**Authors:** Zhenhui Yang, Tiev Miller, Zou Xiang, Marco Y. C. Pang

**Affiliations:** 1 Department of Rehabilitation Sciences, Hong Kong Polytechnic University, 11 Yuk Choi Road, Hung Hom, Hong Kong; 2 Department of Physical Therapy, Guangdong Provincial Work Injury Rehabilitation Hospital, Guangzhou, China; 3 Department of Health Technology and Informatics, Hong Kong Polytechnic University, Hung Hom, Hong Kong

**Keywords:** Neuroscience, Medical research, Biomarkers

## Abstract

This randomized controlled trial aimed to evaluate the effects of different whole body vibration (WBV) frequencies on concentric and eccentric leg muscle strength, bone turnover and walking endurance after stroke. The study involved eighty-four individuals with chronic stroke (mean age = 59.7 years, SD = 6.5) with mild to moderate motor impairment (Fugl-Meyer Assessment lower limb motor score: mean = 24.0, SD = 3.5) randomly assigned to either a 20 Hz or 30 Hz WBV intervention program. Both programs involved 3 training sessions per week for 8 weeks. Isokinetic knee concentric and eccentric extension strength, serum level of cross-linked N-telopeptides of type I collagen (NTx), and walking endurance (6-min walk test; 6MWT) were assessed at baseline and post-intervention. An intention-to-treat analysis revealed a significant time effect for all muscle strength outcomes and NTx, but not for 6MWT. The time-by-group interaction was only significant for the paretic eccentric knee extensor work, with a medium effect size (0.44; 95% CI: 0.01, 0.87). Both WBV protocols were effective in improving leg muscle strength and reducing bone resorption. Comparatively greater improvement in paretic eccentric leg strength was observed for the 30 Hz protocol.

## Introduction

Muscle weakness is a major impairment after stroke^1^ and is associated with various aspects of physical function^2^ and bone tissue integrity^3^. According to a recent systematic review^4^, previous studies involving the use of bone imaging techniques such as peripheral quantitative computed tomography (pQCT)^3,5,6^ and dual-energy X-ray absorptiometry (DXA)^7^ to investigate the impact of stroke on lower limb bone outcomes reported strong associations of muscle strength and mass with bone mineral density and indices of bone strength. Previous work has also demonstrated an increased rate of bone resorption in people with stroke, which was correlated with lower hip bone density^8,9^. Therefore, effective interventions that target muscle strength and bone health are important for stroke rehabilitation.

Whole-body vibration (WBV) augments muscle activation during exercise^10,11^. The mechanical vibration induces reflex muscle activation and increases motor cortex excitability^12,13^. WBV has also been shown to increase peak muscle torque in lower limb muscles^14^, presumably through the recruitment of higher threshold motor units. Improved muscle contractility and force generating capacity have implications for bone health^15^ as muscle contractions provide an important source of dynamic mechanical loading for maintaining bone tissue^15,16^. There is evidence that WBV can reduce the rate of bone resorption in different populations (e.g., post-menopausal women, children with severe motor disabilities, and people with metabolic acidosis)^17–19^.

WBV training has been identified as a potentially viable treatment modality in various patient groups with muscle weakness and consequent bone loss^19–22^, such as people after stroke^3,23–25^. However, research on bone metabolism and muscle strength post-stroke after WBV intervention is scarce and the results are inconclusive^23,24,26^. Thus far, only one study has examined the effects of WBV on bone turnover in people with stroke, and found no significant change in both bone formation and resorption markers following an 8-week WBV intervention (9–15 min, 20–30 Hz)^24^. More research is needed before the use of WBV for modifying bone turnover rate in people with stroke can be considered conclusive. A meta-analyses by Yang et al. demonstrated that the effects of WBV on maximal isometric knee extension strength (5 studies, SMD = 0.23, 95%CI = − 0.27 to 0.74, p = 0.36), and maximal eccentric knee extension strength (2 studies, SMD = 0.09, 95%CI = -0.38 to 0.56, p = 0.71) yielded wide confidence intervals, indicating that the therapeutic value of WBV on improving knee muscle strength post-stroke requires further investigation^26^.

Many factors may account for the discrepancies in results across previous studies in stroke (e.g., sample characteristics, WBV type, WBV frequency, treatment duration, etc.). As various studies differed on multiple factors, it was not feasible to delineate the effects of each factor by comparing the results of different studies. Nevertheless, among these factors, vibration frequency may be a particularly important parameter, as revealed by both animal and human studies. Animal studies have shown that higher frequency WBV can enhance osteogenesis more effectively than relatively lower frequency WBV^27^. In people with stroke, a greater level of leg muscle activation, as indicated by electromyography (EMG) findings, was found during exposure to higher WBV frequency (30 Hz) than lower frequency (20 Hz)^11,28^. Therefore, repeated exposure to WBV of higher frequencies may lead to a greater strengthening effect of the muscles being stimulated. In a randomized controlled trial, Wei et al. showed that when controlling for the total number of vibrations, a 40 Hz frequency WBV protocol led to the best outcomes in terms of muscle size, strength and physical performance (i.e., 10-m walk test, timed-up-and-go, and sit-to-stand) in patients with sarcopenia^29,30^. However, these findings are not necessarily generalizable to individuals with chronic stroke. Stroke-related impairments are heterogeneous in presentation, etiologically complex (compensatory movement patterns, learned disuse, etc.) and are often inconsistent with typical muscle changes and performance deficits associated with atrophy or aging alone^31,32^. Only one study has compared the effects of two different WBV protocols in the same sample of people with stroke (20 Hz vs 30 Hz) and found no difference in knee muscle strength after 10 weeks of intervention. However, the number of vibrations was not controlled and bone turnover was not measured^33^.

To address these identified gaps in knowledge, we aimed to evaluate the effects of different WBV frequencies in stroke patients. In addition to leg muscle strength and bone turnover, the 6-min walk test (6MWT), an indicator of walking endurance, was also used as an outcome. Leg muscle strength has demonstrated a strong association with 6MWT distance in individuals with stroke^34,35^. Therefore, any WBV-induced improvement in leg muscle strength was also thought to result in better walking endurance. We hypothesized that a higher WBV frequency (30 Hz) would induce larger improvements in muscle strength and walking endurance, and greater reduction in the level of bone resorption marker compared with a lower WBV frequency (20 Hz).

## Methods

### Study design

A single-blinded, randomized controlled trial was conducted.

### Ethical approval

This study was registered on 06/11/2019 in clinicaltrials.gov (identifier: NCT03982251). Ethical approval for the study was granted by the Human Research Ethics Subcommittee of the Hong Kong Polytechnic University (reference number: HSEARS 20140226001-03), and all of the experiments were conducted in accordance with the Declaration of Helsinki. Written informed consent was obtained from each participant prior to data collection.

### Participants

This study was conducted in a research laboratory at the University. Participants were recruited from a stroke patient organization in the community via convenience sampling. The screening and enrolment of potential participants were performed by an independent researcher.

The inclusion criteria were as follows: (1) patient aged ≥ 50 years, (2) medically stable, (3) able to stand for at least 1 min with hand support, and (4) able to understand simple verbal commands. Only individuals aged 50 years or more were recruited. It was because stroke is more prevalent in older adults^36^. Setting an age limit would make the sample more homogeneous in terms of age thereby reducing the potential confounding effect of age on primary outcomes (i.e., muscle strength, bone turnover). The exclusion criteria were as follows: (1) additional neurological conditions, (2) musculoskeletal conditions affecting leg muscle performance (e.g., rheumatoid arthritis), (3) presence of metal implants in the lower extremity, (4) recent fracture in the lower extremity (within 1-year post-onset), (5) receiving medications to treat osteoporosis, (6) vestibular disorders, (7) peripheral vascular disease, and (8) other serious illnesses or contraindications to exercise.

### Participant allocation

**Figure 1:**
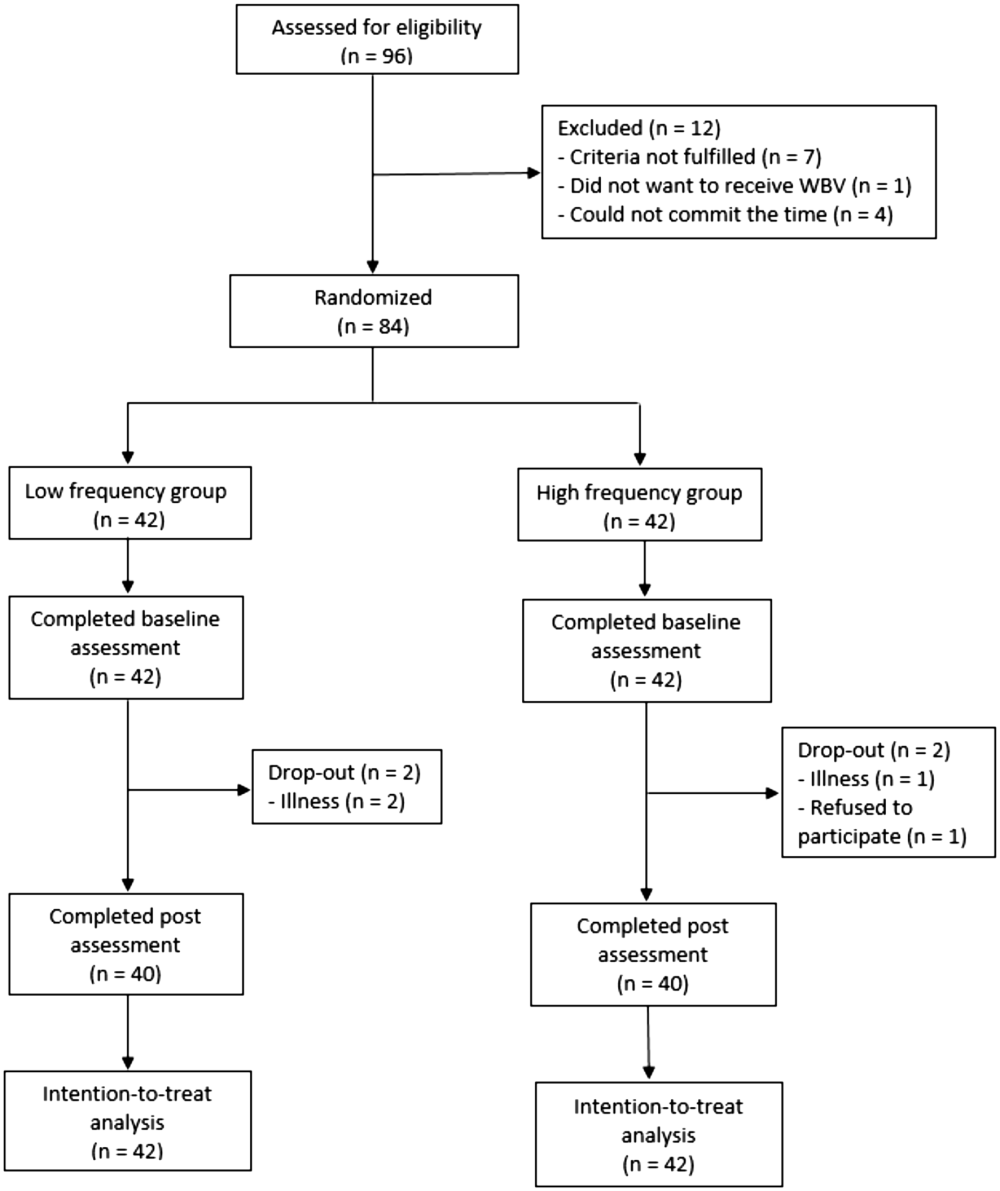
CONSORT flow diagram.

The participants were randomly allocated to one of two groups: a low frequency WBV group (frequency: 20 Hz; amplitude: 0.60 mm) or a relatively higher frequency WBV group (frequency: 30 Hz; amplitude: 0.60 mm). The allocation (with a 1:1 ratio) was completed by an off-site researcher who was not involved in other aspects of the trial, using an online randomization program (http://rct.mui.ac.ir/q/). Prior to randomization, participants were stratified into three clinically meaningful groups according to walking speed (household ambulators: < 0.4 m/s; limited community ambulators: 0.4–0.8 m/s; community ambulators: > 0.8 m/s) and sex^37^. These variables were used for stratification because they were shown to be associated with muscle strength and bone status^3,34,38–41^. The stratified random allocation would ensure that the 20 Hz and 30 Hz groups were similar in terms of walking function and proportion of men/women. The reporting of results and procedures was done in accordance with the Consolidated Standards of Reporting Trials (CONSORT) guidelines. A diagram outlining the flow of participant screening, randomization and allocation is provided in Fig. 1.

### Intervention protocol

The two groups of participants completed training sessions 3 days a week for 8 weeks. A make-up session was provided for any missed appointments so that all participants eventually completed 24 training sessions. The duration of the intervention was based on a previously published study that reported a positive effect of a similar training dosage on bone turnover in post-menopausal women^19^. There is no established WBV protocol for enhancing muscle strength and bone health in people with stroke, and no stroke study has specifically examined the effect of WBV interventions involving different frequencies. Stroke patients also share similar bone health problems to those associated with post-menopausal women (i.e., increased rate of bone resorption and compromised bone density). Therefore, it is reasonable to take reference from this study on post-menopausal women when we developed our WBV protocol. WBV-induced changes in muscle strength and bone turnover marker levels were expected to be evident within the 8-week time frame^21,42–45^.

In each training session, the participants first performed warm-up exercises for ~ 10 min, which included general mobilization and upper limb stretching exercises performed in a sitting position. A Jet-Vibe System (Danil SMC Co. Ltd., Seoul, Korea) was then used to deliver the WBV. This device delivers synchronous vertical vibrations over a range of frequencies (20–55 Hz), which were adjusted by the researchers. Synchronous vertical WBV was used because it was thought to provide better stability during WBV exercise as it produces only vertical perturbations in comparison to both vertical and horizontal displacements associated with side-alternating or oscillating WBV^46,47^. Frequencies > 30 Hz were not used. The pilot data showed that high frequencies caused discomfort in this population. Frequencies < 20 Hz were not used due to potential resonance^48^ and sensorimotor coordination effects^49^.

During the WBV treatment, the participants were instructed to remove their shoes and stand on the vibration platform with their feet placed a shoulder-width distance apart. Participants were instructed to flex the knee to 60° while standing on the vibration platform. This specified joint angle was chosen to reduce undesirable transmission of vibration to the head^50,51^. Based on the results of a previous study, knee extensor muscle EMG activity during WBV exercise was shown to be greatest at 60° of knee joint flexion compared to 10° and 30°^11^. This angle was also determined to be safe and feasible during pilot testing. Participants were also asked to report any symptoms of pain or abnormal discomfort during WBV sessions. To facilitate a meaningful comparison and to delineate the effects of the WBV frequency, the number of loading cycles was matched between the 20 Hz and 30 Hz frequency groups. For both groups, exposure to vibration was provided in 1-min bouts, with a 1-min rest period between bouts. Twelve WBV bouts were delivered per training session to the 20 Hz frequency group, whereas 8 WBV bouts were delivered per training session to the 30 Hz frequency group (i.e., 14,400 loading cycles) so that the total WBV dosage for each session was equivalent between groups. For standardization, all participants gently held onto the handrail of the WBV device only to maintain balance.

### Outcome measures

Researchers who were blinded to the intervention groups conducted all of the outcome assessments. Relevant demographic information and clinical history were obtained from all participants through interviews conducted at baseline. During the baseline assessment session, the level of motor impairment of the leg and foot was evaluated using the Fugl-Meyer Motor Assessment (FMA)^52^. The Physical Activity Scale for the Elderly (PASE)^53^ was used to measure participant physical activity level. The spasticity of the paretic ankle joint was examined using the Modified Ashworth Scale (MAS)^54^. All of the following outcomes were assessed at baseline and also the end of the eight-week intervention period.

*Isokinetic knee muscle strength*: Participants underwent knee muscle strength testing on both sides using an isokinetic dynamometer (HUMAC NORM Testing & Rehabilitation System, Computer Sports Medicine Inc., U.S.A.), which provided good reliability of strength measurements (ICC = 0.89–0.96)^55^. In brief, participants maintained an upright sitting position while the knee joint was aligned with the mechanical axis of the dynamometer. Straps were used to stabilize the untested limb. Each participant was then instructed to perform maximal concentric/eccentric knee extension throughout a range of 10°–70° knee flexion on each side at a constant angular speed of 120°/s. This range of motion was chosen based on the experience gained in our pilot testing. Some individuals with stroke patients had limited hamstrings flexibility, and were not able to reach the 0° knee flexion (i.e., full knee extension) in a sitting position. Some individuals experienced some discomfort if the knee (particularly on the paretic side) was flexed to more than 80°, potentially indicative of degenerative joint changes. Therefore, to ensure safety, we used a range of motion between 10° and 70° of knee flexion. The relatively high angular speed of 120°/s was chosen for several reasons. First, it was a speed commonly used in previous stroke studies^56,57^. Second, individuals with stroke typically demonstrated severe muscle weakness at higher contraction speeds^1^. Also, knee movements at high speeds are involved in daily activities. Previous work showed that during walking over a wide range of speeds (0.4–1.39 m/s), the angular velocity during knee flexion in the swing phase, and that of knee extension during terminal swing, exceeded 120°/s^58^. As walking speed approached 1.0 m/s, the angular velocity of knee flexion during the loading response also approximated 120°/s. During the sit-to-stand movement, the knee joint angular velocity has also been shown to be roughly 120°/s during the extension phase^59^. The sequence of testing (i.e., paretic side versus non-paretic side, or type of contraction) was randomized to minimize the order effect. Three trials were recorded for each test condition and the total work (in Joules; J) value was obtained using customized software. Three trials were conducted to obtain the mean value for statistical analysis. The total work represents the accumulated torque output produced as the joint moves through a specified range of motion^60^. Therefore, the measurement of total work takes into account the ability of the muscle to maintain contraction at a certain strength level through the range of motion. The measure was thus considered by some researchers to be more reflective of muscle function and strength during movement than peak torque^61–63^. The percent standard error of measurement (%SEM) established for this outcome was 19.2%, which is an appropriate index for detecting change in a group of people^64^.

*Bone resorption analysis:* Serum cross-linked N-telopeptides of type I collagen (NTx) was chosen as a surrogate marker of bone resorption to evaluate the dynamic process of bone turnover. In brief, a 5 ml fasting blood sample was collected from all participants in the morning (between 0900 and 1100 h) at defined investigational time points. All of the blood samples were then promptly centrifuged. The serum was separated and then immediately frozen at − 80 °C until further analysis. Serum levels of NTx were assessed using the Osteomark NTx Serum assay (Alere Scarborough, Inc., Scarborough, U.S.A.) according to the protocol provided by the manufacturer. Essentially, appropriately diluted serum samples, together with NTx epitope-containing molecules that are conjugated with horseradish peroxidase, were added to the microplate wells that had been previously coated with antibodies against NTx. NTx in the patient sample thus competed with the conjugated NTx epitopes in the microplate well for antibody binding sites. Following a wash step, a chromogenic substrate solution was added for color development. Absorbance was determined on a spectrophotometer and the NTx concentration was calculated against a standard calibration curve. The assay values were recorded in nanomoles Bone Collagen Equivalents per liter (nM BCE). The reference range was between 3.2 and 40.0 nM BCE. In each assay, three duplicate samples were used to determine the intra-assay coefficient of variation (% CV) (intra-assay %CV = 4.6%). The inter-assay %CV was established between two assays of a total of 18 samples (inter-assay %CV = 6.9%). An intra-assay and inter-assay %CV value of less than 10% is considered to be acceptable^65^.

*6MWT:* This test was used to assess endurance^66^. Participants were asked to walk along a 15-m walkway and cover as much distance as possible within 6 min, using walking aids if necessary. The total distance (meters) walked was recorded. The 6MWT has demonstrated excellent reliability (ICC = 0.97–0.99) in assessing walking endurance among individuals with stroke^66^.

### Compliance and adverse events

Participant attendance of the training sessions was recorded by the researcher who supervised the WBV training sessions. Any adverse events reported by the participants or observed by the researcher were also documented. The total time period taken to complete 24 training sessions (number of days) and the maximum time lapse between any two training sessions (number of days) for each participant were used for subsequent analysis.

### Statistical analyses

The sample size was estimated using G*Power 3.1.9.2 software (Heinrich-Heine-Universität Düsseldorf, Germany). Tihanyi et al. found that WBV induced a significant increase in paretic knee muscle strength (Cohen’s *d*) of 0.46–0.51 (i.e., medium) in people with stroke^67^. Another study by Wei et al. showed that medium-frequency WBV generated better knee muscle strength outcomes than low-frequency outcomes in patients with sarcopenia (*d* = 0.24)^30^. Another study by Turner et al. showed that a WBV protocol similar to that used in the 30 Hz frequency group induced a significant change in bone resorption marker levels, with a large effect size of 0.96^19^. Overall, a small to medium effect size was assumed (*f* = 0.2) for a 2 × 2 analysis of variance (ANOVA) with repeated measures. With an alpha of 0.05, a power of 90%, and considering an attrition rate of 15%, the minimum sample size required to detect a significant group × time interaction effect was 80 participants (40 per group).

The Statistical Package for Social Sciences version 23.0 (SPSS; IB, Armonk, NY) was used for all analyses. The normality of the data was checked using the Kolmogorov–Smirnov test. Between-group differences in baseline characteristics were evaluated by an independent t test, a Mann–Whitney U test or a chi-square test, as appropriate. To compare the treatment effect between the two groups, a mixed-design, analysis of variance was used (within-subject factor: time; between-subject factor: group). An intention-to-treat analysis was conducted, in which the last observation carried forward method was used to substitute the missing data for participants who were lost to follow-up (i.e. dropout). This approach was considered to be more conservative but was less susceptible to bias arising from attrition^68^. Post-hoc analyses were conducted to examine the pre-test and post-test within-group scores (paired t-tests), and also between-group differences in change scores (independent t-tests). The above analyses were repeated after eliminating drop-outs (on-protocol analysis). If both the intention-to-treat and on-protocol analysis approaches yielded similar findings, there would be strong confidence in the study results^68^.

## Results

### Study group characteristics

Of the 96 individuals with stroke who were screened for eligibility, 84 fulfilled all of the selection criteria. This was greater than the estimated minimum number of participants required from our sample size calculation (n = 80). As having a greater sample size could further increase statistical power, 84 individuals with stroke were ultimately enrolled in the study rather than 80. They were randomly allocated to either the 20 Hz frequency (n = 42) or 30 Hz frequency (n = 42) groups. Four participants dropped out during the course of the study (2 in each of the treatment groups). By the end of the study, 80 participants had completed the training program and all outcome assessments (Fig. 1). We found no significant between-group differences in terms of demographic variables or stroke characteristics at baseline (Table 1). Therefore, none of the variables shown in Table 1 were considered important confounding factors.

**Table 1:** Participant characteristics at baseline^*^.

Characteristic	All (n = 84)	20 Hz WBV (n = 42)	30 Hz WBV (n = 42)	*p*^†^
**Demographics**				
Age (years)	59.7 ± 6.5	60.4 ± 5.9	59.0 ± 7.0	0.299
Sex (men/women)	54/30	29/13	25/17	0.362
Body mass index h(kg/m^2^)	23.5 ± 3.3	23.0 ± 3.1	24.0 ± 3.4	0.203
Walking aid: none/cane/quad/frame	68/13/3/0	35/6/1/0	33/7/2/0	0.791
PASE score	95.1 ± 50.3	97.2 ± 57.0	93.1 ± 43.2	0.715
**Stroke characteristics**				
Hemiparesis side, n (right/left)	37/47	17/25	20/22	0.510
Post-stroke duration (years)	4.6 ± 3.5	4.6 ± 3.7	4.5 ± 3.4	0.951
Type of stroke, n (hemorrhagic/ischemic)	40/44	17/25	23/19	0.190
Fugl-Meyer lower limb score	24.0 ± 3.5	24.6 ± 2.8	23.2 ± 4.1	0.060
Paretic ankle MAS score (0–4)	1.0 (0–4) ^‡^	1.0 (0–4) ^‡^	1.0 (0–4) ^‡^	0.075
**Comorbidities, n**				
Hypertension	54	28	26	0.649
Diabetes mellitus	15	8	7	0.776
Hyperlipidemia	32	17	15	0.653
Number of comorbidities	1.7 ± 0.8	1.6 ± 0.9	1.8 ± 1.0	0.495
**Medications, n**				
Antihypertensives	54	28	26	0.649
Antidiabetic medications	15	8	7	0.776
Anticonvulsants	35	18	17	0.825
Anticoagulants	31	16	15	0.821
Number of medications	3.0 ± 1.8	3.1 ± 1.7	2.9 ± 1.8	0.462
**Compliance**				
Time taken to complete 24 training sessions (d)	65.4 ± 3.1	65.3 ± 3.2	65.5 ± 3.1	0.784
Maximum time lapse between training sessions (d)	8.0 ± 2.2	7.8 ± 2.3	8.1 ± 2.1	0.527

*Mean ± standard deviation presented unless indicated otherwise.

^†^Between-group comparison.

^‡^Median (interquartile range).

Abbreviations: 20 Hz WBV: 20 Hz whole-body-vibration group, 30 Hz WBV: 30 Hz whole-body-vibration group, PASE: Physical Activity Scale for the Elderly, MAS: Modified Ashworth Scale.

### Effect on outcome measures

The outcome measurements collected at baseline did not differ between the two groups (Table 2). We identified a significant main effect of time for all muscle strength and bone turnover outcomes (p < 0.001), but not for the 6MWT (p = 0.533) (Table 2). We also identified a significant effect of time × group interaction for the paretic eccentric knee extensor work, with a medium effect size (0.44; 95% CI: 0.01, 0.87). The change of eccentric extensor work in the non-paretic leg also showed a similar trend, but the confidence intervals suggested a small chance that the 20 Hz frequency protocol might be superior (95% CI: -0.08, 0.78). The on-protocol analysis generated similar results (Supplementary Table).

**Table 2:** Outcome measurements (Intention-to-treat analysis).

Variable	20 Hz WBV (N = 42)			30 Hz WBV (N = 42)			Between-group difference in change scores	Comparisons					
	Pre	Post	Change score	Pre	Post	Change score	Mean (95% CI)	*p*^*a*^	*p*^*b*^	*p*^*c*^	*p*^*d*^	*p*^*e*^	*p*^*f*^
**Knee extensor work (J)**													
Nonparetic concentric*^‡§^	28.1 ± 11.1	35.8 ± 17.4	7.8 ± 12.4	23.5 ± 11.1	36.3 ± 23.4	12.8 ± 17.6	5.0 (− 1.6, 11.7)	0.061	0.000	0.135	0.000	0.000	0.135
Paretic concentric*^‡§^	17.4 ± 8.0	22.4 ± 9.0	5.0 ± 5.1	15.3 ± 8.2	21.0 ± 10.0	5.7 ± 4.8	0.7 (− 1.4, 2.9)	0.242	0.000	0.496	0.000	0.000	0.496
Nonparetic eccentric*^‡§^	80.3 ± 20.4	94.5 ± 22.9	14.2 ± 13.4	75.1 ± 19.9	95.5 ± 30.2	20.4 ± 21.0	6.2 (− 1.5, 13.8)	0.243	0.000	0.113	0.000	0.000	0.113
Paretic eccentric*^†‡§¶^	65.2 ± 21.2	73.0 ± 18.7	7.8 ± 11.2	60.2 ± 23.3	74.0 ± 21.6	13.8 ± 15.5	5.9 (0.05, 11.8)	0.311	0.000	0.048	0.000	0.000	0.048
**Other outcomes**													
NTx (nM BCE)*^‡§^	6.1 ± 3.7	3.6 ± 2.3	− 2.2 ± 3.4	6.3 ± 4.2	3.6 ± 1.8	− 2.7 ± 4.0	− 0.5 (− 2.1, 1.1)	0.792	0.000	0.540	0.000	0.000	0.540
6MWT distance (m)	248.7 ± 89.8	250.6 ± 90.3	1.9 ± 11.9	257.2 ± 111.4	256.8 ± 112.5	− 0.4 ± 11.0	− 2.3 (− 7.3, 2.7)	0.701	0.533	0.365	0.302	0.835	0.365

*Significant time effect (*p* < 0.05).

^†^Significant group × time interaction effect (*p* < 0.05).

^‡^Significant within-group comparison (20 Hz WBV group) (*p* < 0.05).

^§^Significant within-group comparison (30 Hz WBV group) (*p* < 0.05).

^¶^Between-group comparison of change score (*p* < 0.05).

^a^Baseline comparisons (independent t-test).

^b^Time effect (ANOVA).

^c^Group × time interaction effect (ANOVA).

^d^Within-group comparison (20 Hz WBV group) (paired t-test).

^e^Within-group comparison (30 Hz WBV group) (paired t-test).

^f^Between-group comparison of change score (independent t-test).

Abbreviations: 20 Hz WBV: 20 Hz whole-body-vibration group, 30 Hz WBV: 30 Hz whole-body-vibration group, CI: confidence interval, NTx: serum cross-linked N-telopeptides of type I collagen, 6MWT: 6-min walk test.

### Compliance and adverse events

The time taken to complete 24 training sessions and the maximum time lapse between any two training sessions were similar between the two groups (p > 0.05). No adverse events occurred during the intervention trial.

## Discussion

The key finding is that both the 20 Hz and 30 Hz WBV protocols are effective in increasing knee muscle strength and reducing bone resorption, but the former is better at improving the paretic eccentric knee extensor strength than the latter.

Leg muscle activity measured by EMG can be augmented during WBV exposure^69–71^. Through regular WBV intervention, the stimulated muscles are repetitively “exercised”. Over time, this may contribute to greater muscle strength^15^. Our study data confirm that leg muscle strength can be increased following regular WBV intervention over an 8-week period. Improved muscle coordination^67^, and enhancement of intramuscular blood perfusion^72^ are some of the proposed mechanisms underlying improved muscle strength following WBV reported in studies involving neurological populations. Other mechanisms associated with improved strength following WBV reported in healthy subjects include increased cortical excitability^12^, reduced recruitment threshold, increased activation of fast-twitch muscle motor units^73^, and motor unit reflex activation^47^. Overall, our findings are largely in line with previous studies showing increased muscle strength in elderly populations, both with and without sarcopenia, following WBV exercise^29,74^. However, the positive improvement in muscle strength reported in this study cannot be attributed to the WBV stimulation alone. The participants assumed a static, semi-squatting posture (i.e., 60° of knee flexion) during WBV exposure which may have also contributed to the observed increase in muscle strength.

An interesting finding of our study is that the 30 Hz frequency protocol induced a greater gain in eccentric knee extension strength in the paretic leg. The mean increase in eccentric knee strength attained by the 30 Hz frequency group was 22.9%, which exceeded the %SEM value (i.e. 19.2%). Our results thus suggest the 30 Hz frequency protocol produced a clinically meaningful change in muscle strength. These results are accordant with a previous study that found a 30 Hz WBV frequency to be optimal for improving muscle strength among healthy adults^75^. Compared to relatively lower (20 Hz) and higher WBV frequencies (60 Hz), Wei et al. found that a 40 Hz frequency produced the largest improvement in isokinetic knee extension strength among older adults with sarcopenia^30^. Therefore, it seems that specific frequencies for producing optimal outcomes are different for various patient populations.

In our study, the eccentric strength results for the non-paretic leg showed a similar trend to the paretic leg. However, the confidence intervals (-0.08, 0.78) indicate a slight probability that the 20 Hz frequency protocol might be superior to the 30 Hz frequency protocol. Baseline paretic muscle strength was also substantially lower than the non-paretic side (Table 2), suggesting there was more room for improvement in the former.

Only one randomized controlled study by Liao et al. has attempted to compare WBV protocols for stroke patients^33^. However, the addition of low-intensity (peak acceleration: 1.6 g) high-intensity (3.6 g) WBV in the exercise protocol used in their trial did not improve concentric or eccentric muscle strength outcomes^33^. Differences in the WBV protocol [i.e., smaller (10°) knee flexion angle during WBV exercise, lower number of total bouts per session (2 for 20 Hz, 3 for 30 Hz), longer bout duration (1.5 min), and higher vibration amplitude (1 mm) used in the study by Liao et al.] may provide an explanation for this finding. The authors also compared WBV intensities but were unable to delineate the effect of frequency because of differences in the number of loading cycles involved in different treatment arms^33^.

Both groups improved in concentric muscle strength on both sides after the intervention period but no significant between-group differences were found. Concentric muscle strength is typically more compromised than eccentric muscle strength after stroke^1^, and may require more intensive training to elicit a more pronounced difference in improvements between the two WBV protocols. This theory now requires further research.

Our results show that both the 20 Hz and 30 Hz frequency protocols promoted a significant reduction in the expression of NTx indicating that WBV exercise was beneficial in reducing the rate of bone resorption among chronic stroke patients. The amount of reduction in NTx was similar between the two groups (20 Hz frequency: 36%, 30 Hz frequency: 43%) and was not statistically significant. Perhaps a larger differential in WBV frequency and a larger sample size would be required to detect a significant between-group difference in reduction of NTx levels. Only one previous study has investigated the effects of WBV on bone turnover (indicated by C-telopeptide of type I collagen cross linking and bone-specific alkaline phosphatase levels) in people with stroke^24^. The level of these bone turnover markers showed no significant change after the 8-week intervention period (24 sessions) for both the WBV and control groups. The disparity in results may be related to the difference in WBV protocols^24^. In the previous study, the number of loading cycles was gradually increased as the training program progressed, and did not reach a level similar to the present study until week 5. The less intensive WBV stimulation in the initial period of the intervention program may partly explain why no significant change in bone resorption marker level was reported in their study^24^. As there was no sham WBV or no-intervention control group in this study, the difference in the change of NTx levels after WBV intervention versus a sham intervention is unknown. Previous work has shown that individuals with stroke have a higher level of bone resorption than their counterparts without a history of stroke^8^. Longitudinal studies are required to examine the temporal changes in bone resorption marker levels.

The two treatment protocols did not induce any significant change in 6MWT distance. Apart from muscle strength, aerobic capacity has also been identified as a limiting factor related to 6MWT performance (i.e., walking endurance) among people with stoke^34,35,76,77^. Previous results indicated that exposure to WBV produced only modest changes in cardiovascular parameters, such as heart rate and blood pressure^78^. This may explain, in part, why our intervention protocols did not lead to any significant change in 6MWT distance.

This study has several limitations that should be noted. First, the findings cannot be generalized to those who are in the acute or sub-acute stages of stroke recovery, or to those who are wheelchair-bound or have severe motor impairments. Second, while it is unlikely that age is an important confounding factor in this study due to the lack of significant between-group difference (Table 1), how younger stroke patients might respond to the two WBV frequencies remains to be investigated. Third, we did not measure the level of bone formation markers. Incorporating a bone formation maker in our study would have provided a more comprehensive evaluation of the effect of our WBV protocols on bone turnover. Fourth, only synchronous vertical vibrations were used in our study. It may not be meaningful to make a direct comparison between the results of this study with others using oscillating (side-alternating) vibrations as other factors also differed across studies (i.e., participant demographics, body position, external load)^46^. Whether using side-alternating vibrations may result in greater muscle strength improvement in stroke patients will require further investigation. Finally, whether the beneficial effects can be sustained after the cessation of treatment also remains to be determined.

The results showed that while both the 20 Hz and 30 Hz WBV frequency protocols increased concentric and eccentric knee muscle strength and reduced bone resorption rate, the 30 Hz frequency protocol was more effective than the 20 Hz frequency protocol in improving eccentric knee extension strength on the paretic side after treatment cessation. Therefore, a frequency of 30 Hz may be more appropriate for enhancing leg muscle strength, with possible implications for maintaining bone health among individuals with chronic stroke.

## Supplementary Information

Supplementary Information.

## Data Availability

All data generated or analyzed during this study are included in this published article and its Supplementary Information files.
