# Supplementary material for: Effects of different vibration frequencies on muscle strength, bone turnover and walking endurance in chronic stroke
Source: Sci Rep. 2021 Jan 8;11:121. doi: 10.1038/s41598-020-80526-4 (PMC7794423; doi:10.1038/s41598-020-80526-4)
Supplement: Supplementary file 1 — Supplementary Information. [file 41598_2020_80526_MOESM1_ESM.pdf]

Effects of different vibration frequencies on muscle strength, bone turnover and walking endurance in chronic stroke

Authors: Zhenhui Yang, Tiev Miller, Zou Xiang, Marco Y. C. Pang

Supplemental Table. Outcome measurements (On-protocol analysis)

Variable	20Hz WBV (N = 40)			30Hz WBV (N = 40)			Between-group difference in change scores	Comparisons					
	Pre	Post	Change score	Pre	Post	Change score	Mean (95% CI)	p^a	p^b	p^c	p^d	p^e	p^f
**Knee extensor work (J)**													
Nonparetic concentric*‡§	28.3±10.9	36.4±17.4	8.2±12.6	24.0±11.0	37.4±23.4	13.4±17.8	5.3 (-1.6, 12.2)	0.084	0.000	0.131	0.000	0.000	0.131
Paretic concentric*‡§	17.6±8.2	22.9±8.9	5.3±5.1	15.5±8.2	21.6±9.8	6.0±4.8	0.78 (-1.4, 3.0)	0.260	0.000	0.483	0.000	0.000	0.483
Nonparetic eccentric*‡§	80.8±20.6	94.5±22.9	14.9±13.4	75.8±20.1	95.5±30.2	21.4±21.0	6.5 (-1.4, 14.3)	0.273	0.000	0.104	0.000	0.000	0.104
Paretic eccentric*†‡§¶	65.4±21.5	73.0±18.7	8.2±11.4	61.0±23.2	74.0±1.6	14.5±15.6	6.2 (0.16, 12.29)	0.377	0.000	0.044	0.000	0.000	0.044
**Other Outcomes**													
NTx (nM BCE)*‡§	5.9±3.7	3.6±2.3	-2.3±3.4	6.4±4.3	3.6±1.8	-2.8±4.0	-0.5 (-2.2, 1.2)	0.607	0.000	0.535	0.000	0.000	0.535
6MWT distance (m)	249.7±92.0	251.7±92.4	2.0±12.2	262.0±111.2	261.7±112.4	-0.4±11.3	-2.4 (-7.6, 2.8)	0.590	0.533	0.365	0.302	0.835	0.365

*Significant time effect ($p<0.05$)
†Significant group × time interaction effect ($p<0.05$)
‡Significant within-group comparison (20Hz WBV group) ($p<0.05$)
§Significant within-group comparison (30Hz WBV group) ($p<0.05$)

[¶]Between-group comparison of change score ($p<0.05$)
[a]Baseline comparisons (independent t-test)
[b]Time effect (ANOVA)
[c]Group × time interaction effect (ANOVA)
[d]Within-group comparison (20Hz WBV group) (paired t-test)
[e]Within-group comparison (30Hz WBV group) (paired t-test)
[f]Between-group comparison of change score (independent t-test)
Abbreviations: 20Hz WBV: 20Hz whole-body-vibration group, 30Hz WBV: 30Hz whole-body-vibration group, CI: confidence interval, NTx: serum cross-linked N-telopeptides of type I collagen, 6MWT: 6-minute walk test
